# Generation of a *TP53*-modified porcine cancer model by CRISPR/Cas9-mediated gene modification in porcine zygotes via electroporation

**DOI:** 10.1371/journal.pone.0206360

**Published:** 2018-10-23

**Authors:** Fuminori Tanihara, Maki Hirata, Nhien Thi Nguyen, Quynh Anh Le, Takayuki Hirano, Tatsuya Takemoto, Michiko Nakai, Dai-ichiro Fuchimoto, Takeshige Otoi

**Affiliations:** 1 Laboratory of Animal Reproduction, Faculty of Bioscience and Bioindustry, Tokushima University, Tokushima, Japan; 2 Division of Embryology, Institute of Advanced Medical Sciences, Tokushima University, Tokushima, Japan; 3 Division of Animal Sciences, Animal Biotechnology Unit, Institute of Agrobiological Sciences, National Agriculture and Food Research Organization (NARO), Ibaraki, Japan; Friedrich-Loeffler-Institute, GERMANY

## Abstract

*TP53* (which encodes p53) is one of the most frequently mutated genes in cancers. In this study, we generated *TP53-*mutant pigs by gene editing via electroporation of the Cas9 protein (GEEP), a process that involves introducing the Cas9 protein and single-guide RNA (sgRNA) targeting exon 3 and intron 4 of *TP53* into *in vitro*-fertilized zygotes. Zygotes modified by the sgRNAs were transferred to recipients, two of which gave birth to a total of 11 piglets. Of those 11 piglets, 9 survived. Molecular genetic analysis confirmed that 6 of 9 live piglets carried mutations in *TP53*, including 2 piglets with no wild-type (WT) sequences and 4 genetically mosaic piglets with WT sequences. One mosaic piglet had 142 and 151 bp deletions caused by a combination of the two sgRNAs. These piglets were continually monitored for 16 months and three of the genome-edited pigs (50%) exhibited various tumor phenotypes that we presumed were caused by *TP53* mutations. Two mutant pigs with no WT sequences developed mandibular osteosarcoma and nephroblastoma. The mosaic pig with a deletion between targeting sites of two sgRNAs exhibited malignant fibrous histiocytoma. Tumor phenotypes of *TP53* mosaic mutant pigs have not been previously reported. Our results indicated that the mutations caused by gene editing successfully induced tumor phenotypes in both *TP53* mosaic- and bi-allelic mutant pigs.

## Introduction

People are living longer than ever; therefore, the number of cancer patients is expected to increase as a result of the high incidence of cancer later in life. Despite the importance of small rodents, such as mice and rats, for preclinical drug studies, these taxa are limited by their considerable differences from humans (e.g., differences in body size, general physiology, anatomy, and lifespan). Therefore, alternative genetically defined cancer models are needed, including larger, longer-lived species that can be monitored in real time under conditions relevant to human patients.

The pig is a useful model for studying human diseases [1–3] and is expected to play a major role in oncology research as a translational model for the development of tumor therapies. However, spontaneous cancers are rare in pigs, with similar rates as observed in humans [4]. A variety of strategies have been implemented to establish porcine cancer models [5–9]. To develop laparoscopic nephrectomy, a pig model of exophytic kidney tumors has been generated by the injection of liquid plastic into the kidney [8]. The artificial injection of chemical carcinogens can also induce tumors, and *N*-Nitrosodiethylamine has been used to induce pig liver hepatocellular carcinoma [9]. Pigs implanted with human tumor cells serve as xenograft tumor models, e.g., an intraperitoneal tumor model [5] and glioblastoma model [6]. Moreover, porcine cells genetically converted to a tumorigenic state and transferred back into the donor pig successfully induced tumorigenesis (autologous transplantation model) [7]. However, these procedures have limited applications and do not fully replicate human cancers.

Genetically modified pigs targeting cancer-specific genes have been established as porcine cancer models, which are expected to replicate human cancers (e.g., *GLI2* gene for basal cell carcinoma [10], *adenomatous polyposis coli (APC)* gene for colorectal cancer [11, 12], and *breast cancer associated gene 1 (BRCA1)* for breast cancer [13, 14]). The transcription factor p53, encoded by *TP53*, is a vital tumor suppressor that promotes senescence or apoptosis following DNA damage induced by cell stress. Mutations in p53 are frequently associated with human cancers [15], and germline mutations in *TP53* are responsible for Li Fraumeni multiple cancer syndrome [16]. Studies of *TP53* have benefited from genetically modified mice, and *TP53* mutant mice have been established as cancer models [17]. Conversely, somatic cell nuclear transfer (SCNT) is the main technique used to generate genetically modified pigs from genetically modified somatic cells for biomedical applications [2, 18]. The number of genetically modified pigs that replicate human diseases has dramatically increased [19]. Several types of *TP53*-modified pigs have been generated using the SCNT technique [20–24] and they successfully developed tumors. Recent, genome editing approaches, such as the clustered regularly interspaced short palindromic repeats (CRISPR)/CRISPR-associated gene (CRISPR/Cas) system [25, 26] have improved the efficiency of generating modified pigs. We used gene editing via electroporation of the Cas9 protein (GEEP) [27], a method in which CRISPR/Cas9 is introduced into pig zygotes by electroporation, to efficient disrupt the targeted gene. In this study, we used GEEP to generate *TP53* mutant pigs and evaluated the resulting pigs as cancer models.

## Results

### Design of sgRNAs and evaluation of the gene editing efficiency

First, we evaluated several single-guide RNAs (sgRNAs) targeting *TP53*. We introduced the Cas9 protein with sgRNAs into *in vitro*-fertilized zygotes by electroporation. After *in-vitro* culture for 7 days, we evaluated the frequencies of mutations in the *TP53* target region of blastocysts. We confirmed that two types of sgRNAs, sgRNA1 and sgRNA2, targeted *TP53* exon 3 and intron 4, respectively (S1 Fig), leading to highly efficient gene editing. The blastocyst formation rates for electroporated zygotes were unaffected by these two sgRNAs (S1 Fig). All fifteen (100%) blastocysts with sgRNA1 and eight of eleven (72.7%) blastocysts with sgRNA2 carried mutations in the *TP53* target region (S1 Fig). The mutation efficiency of blastocysts, determined using the TIDE (Tracking of Indels by DEcomposition) bioinformatics package [28], showed that approximately 45% and 33% of blastocysts with sgRNA1 and sgRNA2 carried bi-allelic mutations, respectively (S1 Fig).

### Embryo transfer and analysis of piglet genotypes

**Fig 1:**
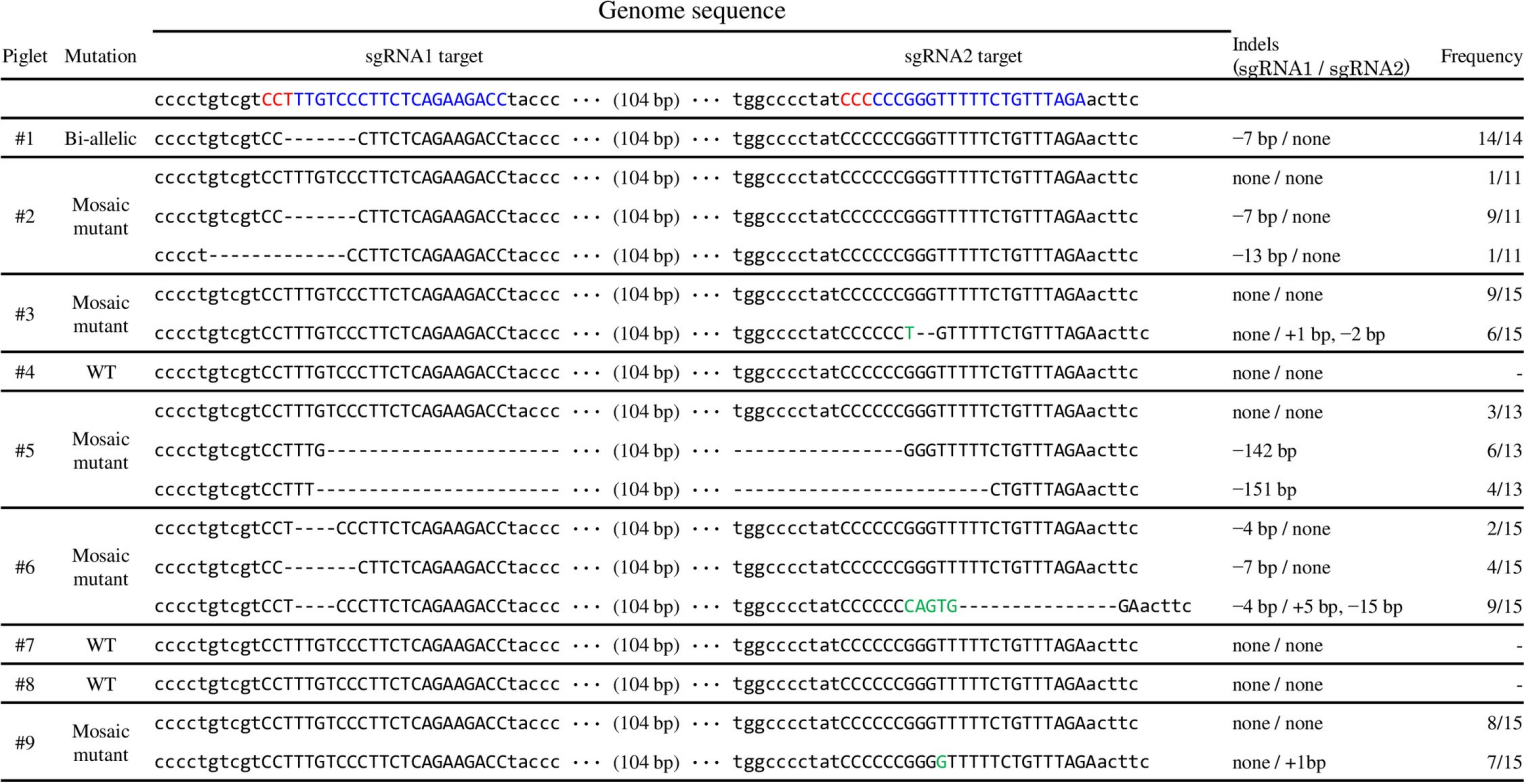
Sequences of the *TP53* target region in 9 genetically modified piglets. The nucleotides in blue and red colors represent target sequences and PAM sequences of each sgRNA, respectively. The nucleotides in green color represent inserted sequences. Frequency was determined by subcloned sequencing analysis. WT: wild-type.

We introduced the Cas9 protein and sgRNAs into *in vitro*-fertilized zygotes by electroporation. Zygotes were electroporated with sgRNA1, sgRNA2, or both of sgRNA1 and sgRNA2, and divided into three groups (Groups 1, sgRNA1; Group 2, sgRNA2; and Group 3, both sgRNA1 and sgRNA2). Electroporation with sgRNA1 (Group 1) was predicted to introduce an insertion or deletion at the targeting site in exon 3. sgRNA2 (Group 2), which was designed to target intron 4, was also used, expecting to introduce a deletion that included the neighboring exon in which the deletion size introduced by CRISPR/Cas9 is generally unpredictable. Electroporation with two sgRNAs (Group 3) was expected to introduce a deletion between exon 3 and intron 4 in the targeting sites of *TP53*. The same number of zygotes from each group were mixed together and transferred into the oviducts of two estrous synchronized-recipient gilts. The two recipients that received the electroporated zygotes became pregnant and gave birth to a total of 11 piglets. Two piglets were crushed by the sow and died 10 days after birth. Genomic DNA of the ear biopsies from remaining live piglets was analyzed to determine whether mutations were introduced into the *TP53* gene and to evaluate the levels of mosaicism. Sequence analysis of the *TP53* genomic regions flanking the target sites revealed that 6 of the 9 live piglets carried mutations in *TP53* (Fig 1). Mosaicism analysis by subcloned sequencing indicated that among the six mutant piglets, two piglets (#1 and #6) had no wild-type (WT) sequences. Piglet #1 carried bi-allelic mutations in the *TP53* gene, but piglet #6 exhibited mosaic genotypes. The other piglets exhibited mosaic genotypes with 40.0% to 90.9% mutations in their genomes. Piglets #1 and #2 carried deletions in exon 3, which was presumed to be induced by sgRNA1. Piglets #3 and #9 carried mutations near the sgRNA2 targeting site in *TP53*. One piglet (#5) had 142 and 152bp deletions that we postulate was caused by the combination of sgRNA1 and sgRNA2. This piglet was presumed to originate from Group 3 zygotes. Piglet #6 carried indels in both regions targeted by sgRNA1 and sgRNA2, which indicates that it also likely originated from Group 3 zygotes. A deletion between targeting sites of sgRNAs was not observed in piglet #6, presumably due to the time lag of the double-strand break (DSB) by sgRNA1 and sgRNA2.

### Tumor phenotypes of TP53-mutant pigs

**Fig 2:**
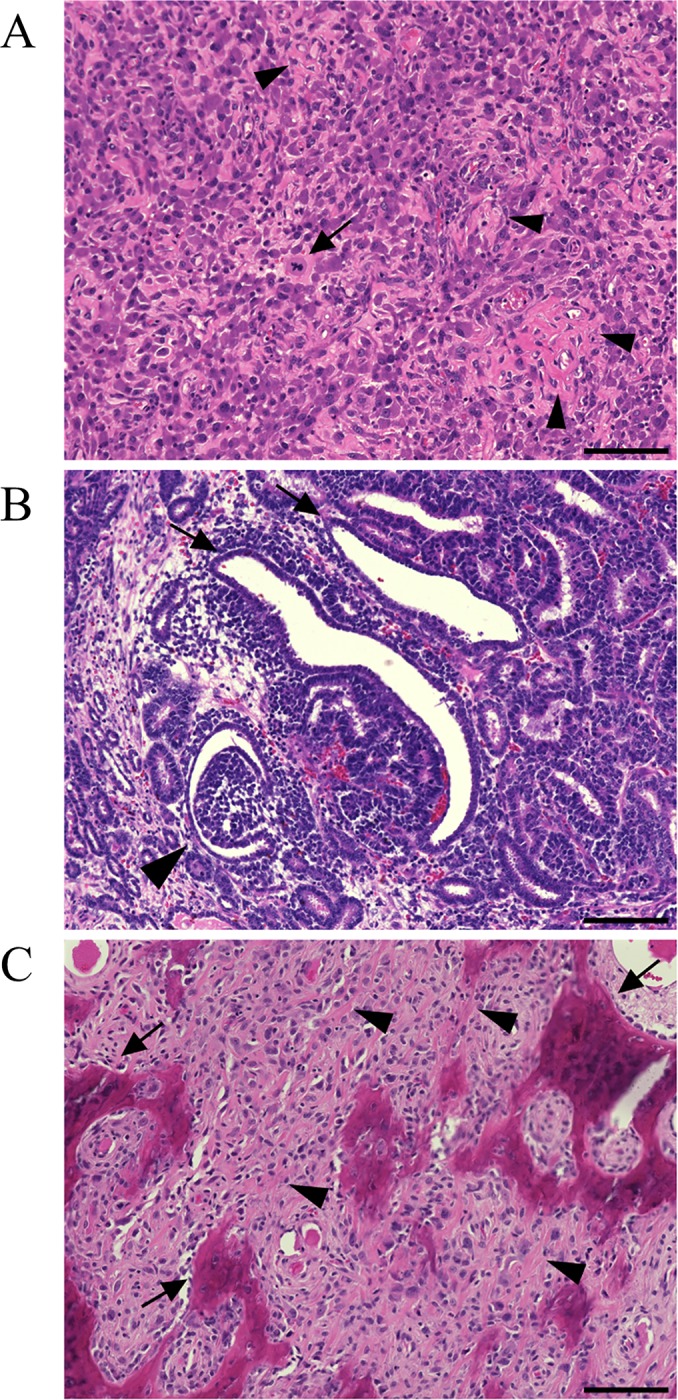
Histopathology of *TP53*-mutant pig tumors stained with hematoxylin and eosin. **A:** Variations in the collagenous fiber levels among tumor cells (arrowheads) of a malignant fibrous histiocytoma were detected in pig #5. Mitotic figures were also observed (arrow). **B:** Nephroblastoma of pig #6. Glomeruloid bodies reminiscent of the fetal nephron (arrowhead) were observed in tumor tissues. Tubular structures mainly composed of neoplastic epithelial cells (arrows) were observed. **C:** Osteosarcoma of pig #1. Pleomorphic non-epithelial cells forming osteoids (arrowheads) and trabecular bone (arrows) in osteosarcoma. The scale bar in each panel (A-C) indicates 100 μm.

The tumor phenotypes of piglets were continuously monitored after birth, and three pigs exhibited multiple tumor phenotypes that we postulate were caused by *TP53* mutations. One mosaic-mutant pig (#5; 12 months old) developed a malignant fibrous histiocytoma in the right front leg that was characterized by frequent mitotic figures, pleomorphic nuclei, spindle-shaped tumor cells, and variations of the collagenous fiber levels among tumor cells (Fig 2A). A large tumor mass was found in the left kidney of pig #6 (14 months old) (S2 Fig). The weight of the right kidney, which was macroscopically normal, was 0.2 kg, whereas that of the left kidney with the tumor mass was 3.14 kg. The mass contained frequent mitotic figures, triphasic (mesenchymal, blastemal, and epithelial) components, and glomeruloid bodies, reminiscent of the fetal nephron in tumor tissues (Fig 2B). Therefore, the tumor was diagnosed as a nephroblastoma. At 16 months after birth, pig #1 showed signs of oral hemorrhaging caused by mandibular osteosarcoma (S2 Fig) that was histologically characterized by pleomorphic non-epithelial cells forming osteoids and trabecular bone, frequent-mitotic figures, and pleomorphic nuclei (Fig 2C). Pig #1 also had a nephroblastoma in the left kidney (S2 Fig). Other organs (e.g., the lung, heart, liver, pancreas, spleen, and kidney) in pigs with tumors were evaluated, and no other types of tumors were detected macroscopically or histologically. The other *TP53*-modified pigs (#2, 3, and 9) and WT (#4, 7, and 8) pigs were sacrificed at 12 to 14 months after birth, and no tumors were detected macroscopically or histologically. Pigs with a tumor phenotype made up 50% (three of six) of the total *TP53*-modified pigs.

### Analysis of tumor genotypes of TP53-mutant pig

**Fig 3:**
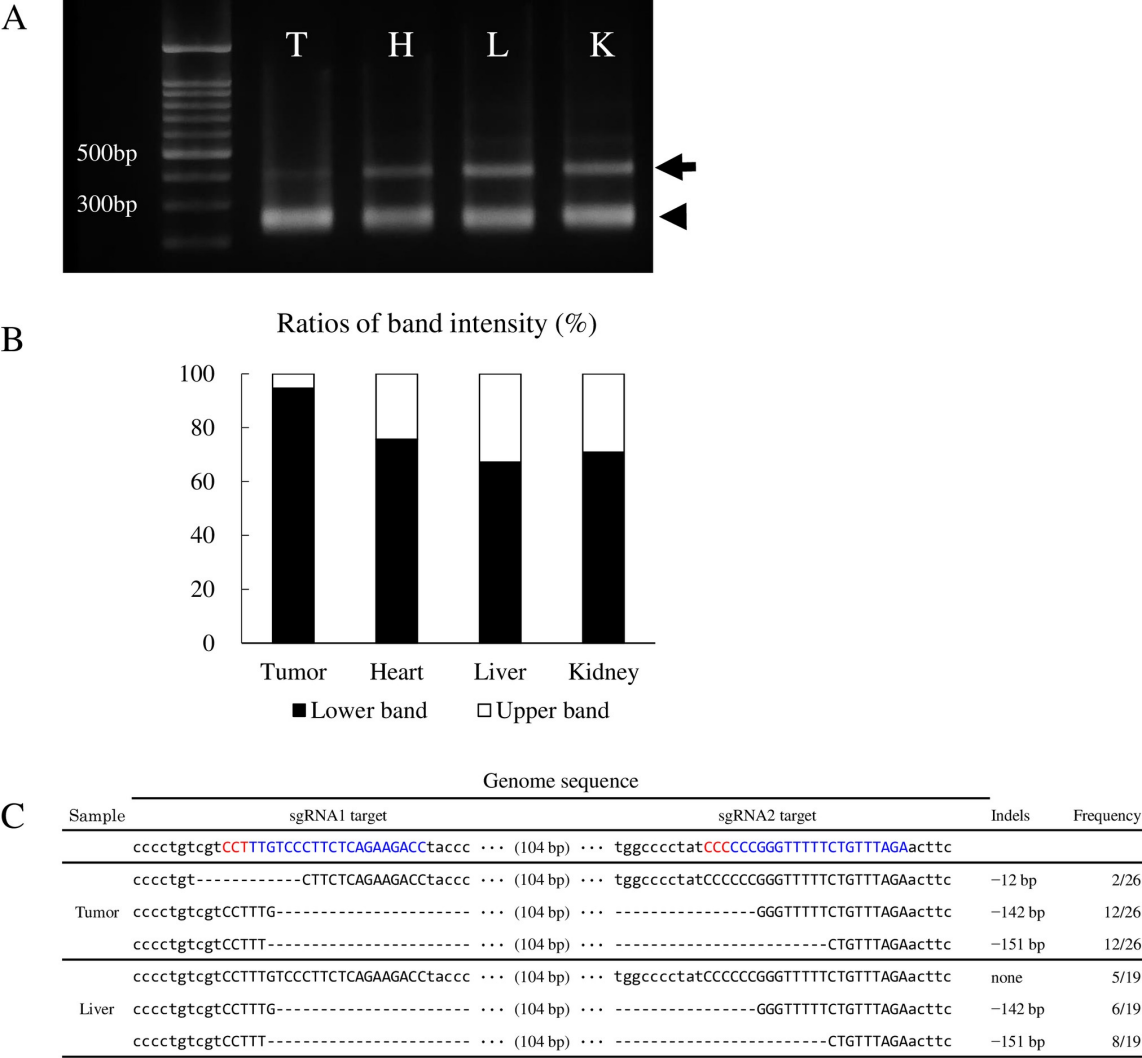
Analysis of tumor tissue genotype collected from *TP53* mosaic pig with wild-type sequences. **A**: Gel image of PCR products of *TP53* from tissues of pig #5. An amplicon of approximately 200bp (arrowhead) indicates mutant allele (142 and 151 bp deletions) which could be distinguished from the wild-type alleles (358bp) and mutant alleles with short indels (arrow). T: tumor tissue, H: heart, L: liver, K: kidney. **B**: The ratio of upper and lower band intensities were quantified using ImageJ software. PCR amplicons from tumor tissues represented a larger frequency of mutant sequences than those from other organs. **C**: Genotype of the *TP53* target region in tumor tissue and liver. The nucleotides in blue and red colors represent target sequences and PAM sequences, respectively. Frequency was determined by subcloned sequencing analysis. WT: wild-type.

Of the three *TP53* mutant pigs with tumor phenotypes, only pig #5 was genetically mosaic with WT sequence. Tissue samples from the tumor, heart, liver, and kidney of pig #5 were amplified by PCR to evaluate the mosaicism of these tissues. Electrophoresis of the amplicons showed two bands, because pig #5 had WT and 142/151bp deleted sequences (Fig 3A). Thus, upper and lower band were predicted to correspond to WT and 142/151bp deleted sequences, respectively. Quantifications of these band intensities using ImageJ software [29] showed that tumor tissue contained a larger amount of mutant amplicons compared to normal tissue samples from the heart, liver, and kidney (Fig 3B). Upper/lower band ratios were comparable between normal tissues (heart, liver and kidney). Next, mosaicism of tumor and liver tissue were compared by subcloned sequencing analysis. Tumor tissue demonstrated that the frequency of mutation was higher than that from liver, and tumor tissue carried no WT sequence (Fig 3C).

## Discussion

Dysfunctional *TP53* is common in cancer; therefore, *TP53*-mutant animals are potentially valuable models to study human cancer. However, *TP53*-modified pigs [20–24] are viable and healthy at an early stage and require long-term monitoring to reveal tumor phenotypes [4, 20]. *TP53* bi-allelic knockout Diannan miniature pigs produced by SCNT did not show tumorigenic signs during survival for <5 months [21]. A recent study has reported that although *TP53* inactivation is sufficient for spontaneous tumorigenesis in pigs, animals younger than 16 months of age show no tumor phenotypes or other abnormalities [22]. Another study reported tumor development in mutant piglets carrying *TP53*^*R167H*^, orthologous to human *TP53*^*R175H*^, which is commonly found in human cancers [24]. In that study, pigs carrying bi-allelic *TP53*^*R167H*^ mutation developed lymphomas as well as osteogenic and renal tumors after reaching sexual maturity, but mono-allelic mutation in *TP53*^*R167H*^ did not lead to tumor development.

In our study, we observed the tumor phenotypes of *TP53* bi-allelic mutant pigs after more than 12 months of monitoring. Among live *TP53*-mutant pigs, three of six *TP53*-mutant pigs (#1, #5 and #6) developed tumor phenotypes. Two of three *TP53*-mutant pigs with tumor phenotype (#1 and #6) carried no WT sequences. Pig #1 carried a bi-allelic mutation in sgRNA1 targeting site. Pig #6 exhibited mosaic genotypes with no WT sequence carrying mutations in both regions targeted by sgRNA1 and sgRNA2, but deletion between targeting sites of two sgRNAs was not observed. We presumed that the lack of deletion between targeting sites of sgRNA1 and sgRNA2 was due to the time lag of the DSB, in which the second DSB by sgRNA might be introduced after the repairment of the first DSB. On the other hand, pig #5 had a mosaic genotype, including WT sequence and deletions between targeting sites of sgRNA1 and sgRNA2. Here, we provide the first report, to our knowledge, of the tumor phenotypes of *TP53* mosaic mutant pigs that include WT sequence. Pig #5 also had tumor phenotypes, and its tumor tissue exhibited a mosaic, including no WT sequence. These results indicated that *TP53* mutated cells mainly increased in the tumor. In addition, we observed that the tumor tissue had a minor mutation with 12bp deletion, however the number of mutated cells with this genotype seemed to be small, because the genotype was not detected from ear biopsy and liver tissue.

The tumor phenotypes of three *TP53*-mutant pigs included two cases of nephroblastoma. A survey of slaughtered pigs showed that the frequency of nephroblastoma is typically quite low [30]. Other tumors observed in this study were osteosarcoma and malignant fibrous histiocytoma. The pig that had malignant fibrous histiocytoma exhibited a high level of basic fetoprotein (BFP) (740 ng/ml), a serum marker for general tumors (S1 Table). In pigs, osteosarcoma is also rare [22, 31] and there are few reports of malignant fibrous histiocytoma [32].

*TP53*-mutant pigs have the potential to be an excellent cancer model. However, pigs as a cancer model are limited by the time requirements and cost. In this study, long-term monitoring (12 to 16 months) was required for tumor detection in *TP53*-mutant pigs, and these results are consistent with those of previous studies [4, 22]. The efficient introduction of the CRISPR/Cas9 system in the zygote by GEEP can help reduce the cost of generating *TP53* mutant pigs. However, the genotype of mutants produced by CRISPR/Cas9 often exhibit mosaic patterns, i.e. these mutant embryos are composed of several types of cells with different mutations [27, 33]. Mosaicism that includes WT cells complicates phenotypic analysis. Moreover, the occurrence of genetic mosaicism needs the production of next generation for porcine model. In the present study, however, we found that *TP53* mosaicism also has the potential to induce tumors. One-step generation of *TP53* mutant pigs by GEEP has an advantage of efficient and time-saving production of tumor models.

The establishment of a pig cancer model exhibiting tumorigenic signs over a short time period will provide a powerful resource for preclinical oncology and basic cancer research. For the efficient development of cancers in pigs, mutations targeting other cancer-related genes have been evaluated. *KRAS*, which belongs to the canonical RAS family, is the most frequently mutated proto-oncogene [34], accounting for 90% of cancer-associated mutations [35]. Consequently, *KRAS* mutant pigs are expected to develop tumors similar to *TP53* mutant pigs. Schook et al. [23] generated pigs with inducible oncogenic *KRAS* and dominant-negative *TP53* and found that the inducible expression of these genes was tumorigenic. Therefore, induction of multiple mutations in cancer-related genes could be an effective strategy for establishing a cancer model in large animals.

In conclusion, we generated *TP53*-modified pigs from zygotes subjected to electroporation with the CRISPR/Cas9 system. Half of the *TP53*-modified pigs, which included mosaic- and bi-allelic mutant pigs, exhibited various tumor phenotypes. Our results indicate that *TP53* mosaic mutant pigs can successfully induce tumor phenotypes as well as bi-allelic mutant pigs.

## Materials and methods

### Animals

Animal experiments were approved by Institutional Animal Care and Use Committee of Tokushima University (approval number: T28-21) and Animal Care Committee of the Institute of Agrobiological Sciences, National Agriculture and Food Research Organization (NARO) (approval number: H28-P14). All animal care and experiments, including maintenance and determination of experimental endpoints, were performed in accordance with the Guidelines for Animal Experiments of Tokushima University and Institute of Agrobiological Sciences, NARO. To alleviate the suffering of animals, all surgical operations and procedures were performed under anesthesia. Early euthanasia was performed based on humane endpoints, defined as either refusal of food or water, signs of pain, or loss of body weight presumed to be induced by tumors. In this study, signs of oral hemorrhaging, refusal of food, and poor general health were observed in pigs with tumors. Early euthanasia was performed on these pigs.

### Oocyte collection, in vitro maturation, and fertilization

Pig ovaries were obtained from prepubertal crossbred gilts (Landrace × Large White × Duroc breeds) at a local slaughterhouse. Cumulus–oocyte complexes (COCs) featuring a uniform ooplasm and compact cumulus cell mass were collected from follicles that were 2–6 mm in diameter, and COCs were cultured in maturation medium at 39°C in a humidified incubator containing 5% CO_2_ and 5% O_2_ as described previously [36]. After maturation for 20–22 h, COCs were cultured for 24 h in maturation medium without hormones.

Matured oocytes were subjected to *in vitro* fertilization, as described previously [36]. Briefly, frozen-thawed spermatozoa were transferred into 6 ml of fertilization medium (PFM; Research Institute for the Functional Peptides Co., Yamagata, Japan) and washed by centrifuging at 500 × *g* for 5 min. The pelleted spermatozoa were resuspended in fertilization medium and adjusted to 5 × 10^6^ cells/ml. Next, COCs were transferred to the sperm-containing fertilization medium and co-incubated for 12 h at 39°C under 5% CO_2_. After co-incubation, the inseminated zygotes were denuded from cumulus cells and attached spermatozoa by mechanical pipetting.

### Preparation of sgRNAs and Cas9 protein

pDR274 plasmids carrying the target sequences were constructed by inserting annealed oligonucleotides into the *Bsa*I site. The oligonucleotides presented in S2 Table were purchased from Sigma-Aldrich (St. Louis, MO, USA). After *Dra*I digestion, sgRNAs were synthesized using the MEGAshortscript T7 Transcription Kit (Ambion, Austin, TX, USA), and purified by phenol-chloroform-isoamyl alcohol extraction and isopropanol precipitation. Precipitated RNA was dissolved in Opti-MEM I (Life Technologies, Gaithersburg, MD, USA). RNA was quantified by absorption spectroscopy and agarose-gel electrophoresis and stored at −30°C until use. The Cas9 protein in the Guide-it sgRNA Screening Kit (Takara Bio, Shiga, Japan) was used for electroporation.

### Electroporation

Electroporation was performed as described previously [27]. Briefly, the electrode (LF501PT1-20; BEX, Tokyo, Japan) was connected to a CUY21EDIT II electroporator (BEX) and set under a stereoscopic microscope. Inseminated zygotes were washed with Opti-MEM I solution and placed in a line in the electrode gap, in a chamber slide filled with 10 μl of Opti-MEM I solution containing sgRNA (200 ng/μl) and the Cas9 protein (50 ng/μl) (Takara Bio). After electroporation (5 × 1-ms pulses at 30 V), zygotes were washed with pig zygote medium (PZM-5; Research Institute for the Functional Peptides Co.) and cultured until embryo transfer (for 12 h) or for 3 days. Embryos cultured for 3 days were subsequently incubated in porcine blastocyst medium (PBM; Research Institute for the Functional Peptides Co.) for 4 days. As a control, some of the inseminated zygotes were cultured with PZM-5 and PBM for 7 days without electroporation.

### Analysis of targeted genes after electroporation

Genomic DNA was isolated from blastocysts or tissues of newborn piglets by boiling them in a 50 mM NaOH solution. After neutralization, the genomic regions flanking the sgRNA target sequences were PCR-amplified using specific primers: sgRNA1 and sgRNA2, 5ʹ-CGAACTGGCTGGATGAAAAT-3ʹ (forward) and 5ʹ-CCAGGGTCCAAGGTCATAGA-3ʹ (reverse). The PCR products from blastocysts and tissues of piglets were extracted by agarose gel electrophoresis. The targeted genomic regions of the PCR products extracted from blastocysts were directly sequenced. Sanger sequencing was performed using a BigDye Terminator Cycle Sequencing Kit ver. 3.1 (Thermo Fisher Scientific, Waltham, MA, USA) and ABI 3500 Genetic Analyzer (Applied Biosystems, Foster City, CA, USA). The TIDE bioinformatics package [28] was used to determine the mutation efficiency in blastocysts. The PCR products extracted from tissues of piglets were cloned into the pMD20 (Takara Bio) plasmid. More than sixteen clones per biopsy were picked up randomly, and the targeted genomic regions were sequenced. Blastocysts and piglets that carried no WT sequences and which had the same mutation pattern in both alleles were classified as having bi-allelic mutations. Blastocysts and piglets that carried more than two mutation types or more than one mutation type in combination with the WT sequences were classified as mosaics. Blastocysts and piglets that only carried the WT sequences were classified as WT.

### Embryo transfer

Two recipient gilts, the estrous cycles of which had been synchronized, were prepared for embryo transfer as described previously [37]. Briefly, pregnant crossbred gilts (Landrace × Large White × Duroc breeds) were terminated at E28 to E40 by intramuscular injection of 0.2 mg of cloprostenol. Subsequently, 0.2 mg of cloprostenol and 1000 IU of eCG were injected 24 h after the first cloprostenol injection. Induction of estrus in recipient gilts by intramuscular injection of hCG (1500 IU) was 72 h after the eCG injection. Approximately 72 h after hCG injection, one- to two-cell stage embryos were transferred into the oviducts of a gilt recipient under anesthesia. Anesthesia was performed by intramuscular injection of ketamine and subsequently maintained with continuous inhalation of 2% to 3% isoflurane. Approximately 100 embryos were transferred to each oviduct, resulting in the transfer of ~200 embryos per gilt.

### Histological assessment

After monitoring for 12 to 16 months, the pigs were euthanized by intravenous injection of saturated solution of potassium chloride under anesthesia by isoflurane. Sample tissues were fixed in 4% paraformaldehyde-neutral buffer solution (Wako, Osaka, Japan) and manually embedded in paraffin. The paraffin-embedded sections were prepared and stained with hematoxylin and eosin using conventional techniques.

### Statistical analysis

The percentages of embryos that developed to the blastocyst stage were subjected to arcsine transformation before ANOVA. The transformed data were evaluated using ANOVA, followed by protected Fisher’s least significant difference tests. Analysis was performed in StatView (Abacus Concepts, Berkeley, CA, USA). *P* < 0.05 was considered statistically significant.

## Supporting information

S1 Fig Confirmation of the sgRNA gene-targeting efficiency and generation of *TP53* mutant blastocysts by GEEP. **A:** Genomic structure of the *TP53* locus and sgRNA sequences targeting *TP53* exon 3 and intron 4. The cutting sites of sgRNA1 and sgRNA2 are shown as scissors and a dotted line. **B:** Blastocyst formation rates for electroporated zygotes. For each treatment group, three replicates with 138–149 oocytes per treatment were analyzed. Error bars; means ± SEM are shown. **C:** The frequency of mutations in the *TP53* target region of blastocysts after zygote electroporation with the Cas9 protein and *TP53* sgRNAs (sgRNA1 and sgRNA2) detected in PCR amplicons. The gene editing success rate was defined as the ratio of the number of mutant blastocysts to the total number of blastocysts **(a)**. Mutation efficiencies of blastocysts as determined by TIDE **(b)**. WT: wild-type, Biallelic: bi-allelic mutant, Mosaic: mosaic mutant.
(TIF)

S2 Fig Macroscopy of *TP53*-mutant pig tumors. **A:** Large tumor mass of a nephroblastoma in the left kidney of pig #6 (arrow). L: Left kidney. R: Right kidney. **B:** Mandibular osteosarcoma (arrow) of pig #1. **C:** Nephroblastoma in the left kidney (arrow) of pig #1. L: Left kidney. R: Right kidney.
(TIF)

S1 Table Tumor marker serum levels in *TP53*-mutant pigs. (DOCX)

S2 Table Oligonucleotide sequences used to generate sgRNAs. (DOCX)
