# Supplementary figures and images for: Generation of a TP53-modified porcine cancer model by CRISPR/Cas9-mediated gene modification in porcine zygotes via electroporation
Source: PLoS One. 2018 Oct 23;13(10):e0206360. doi: 10.1371/journal.pone.0206360 (PMC6198999; doi:10.1371/journal.pone.0206360)

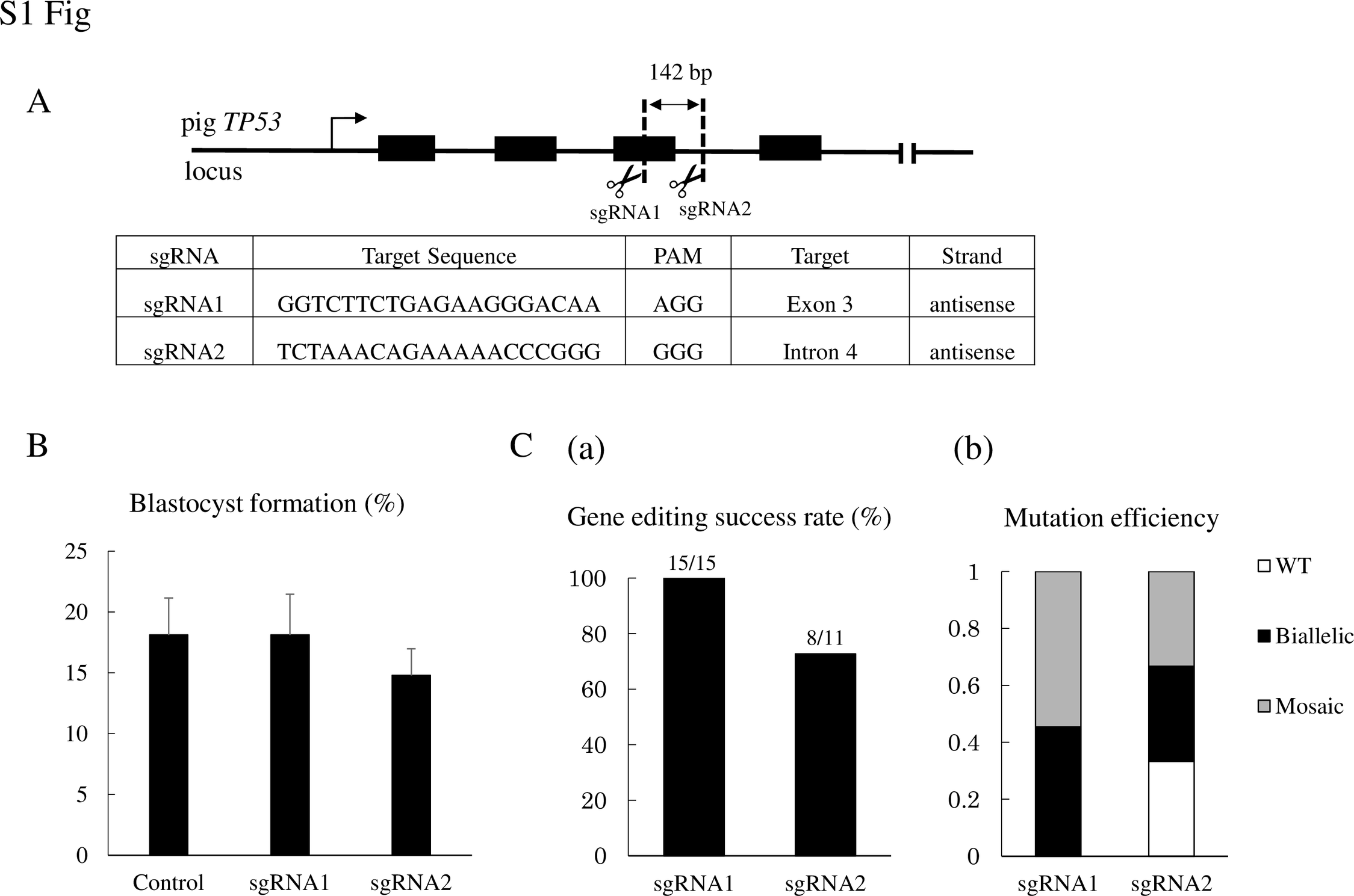

Supplement: S1 Fig — A: Genomic structure of the TP53 locus and sgRNA sequences targeting TP53 exon 3 and intron 4. The cutting sites of sgRNA1 and sgRNA2 are shown as scissors and a dotted line. B: Blastocyst formation rates for electroporated zygotes. For each treatment group, three replicates with 138–149 oocytes per treatment were analyzed. Error bars; means ± SEM are shown. C: The frequency of mutations in the TP53 target region of blastocysts after zygote electroporation with the Cas9 protein and TP53 sgRNAs (sgRNA1 and sgRNA2) detected in PCR amplicons. The gene editing success rate was defined as the ratio of the number of mutant blastocysts to the total number of blastocysts (a). Mutation efficiencies of blastocysts as determined by TIDE (b). WT: wild-type, Biallelic: bi-allelic mutant, Mosaic: mosaic mutant. (TIF) [file pone.0206360.s001.tif]

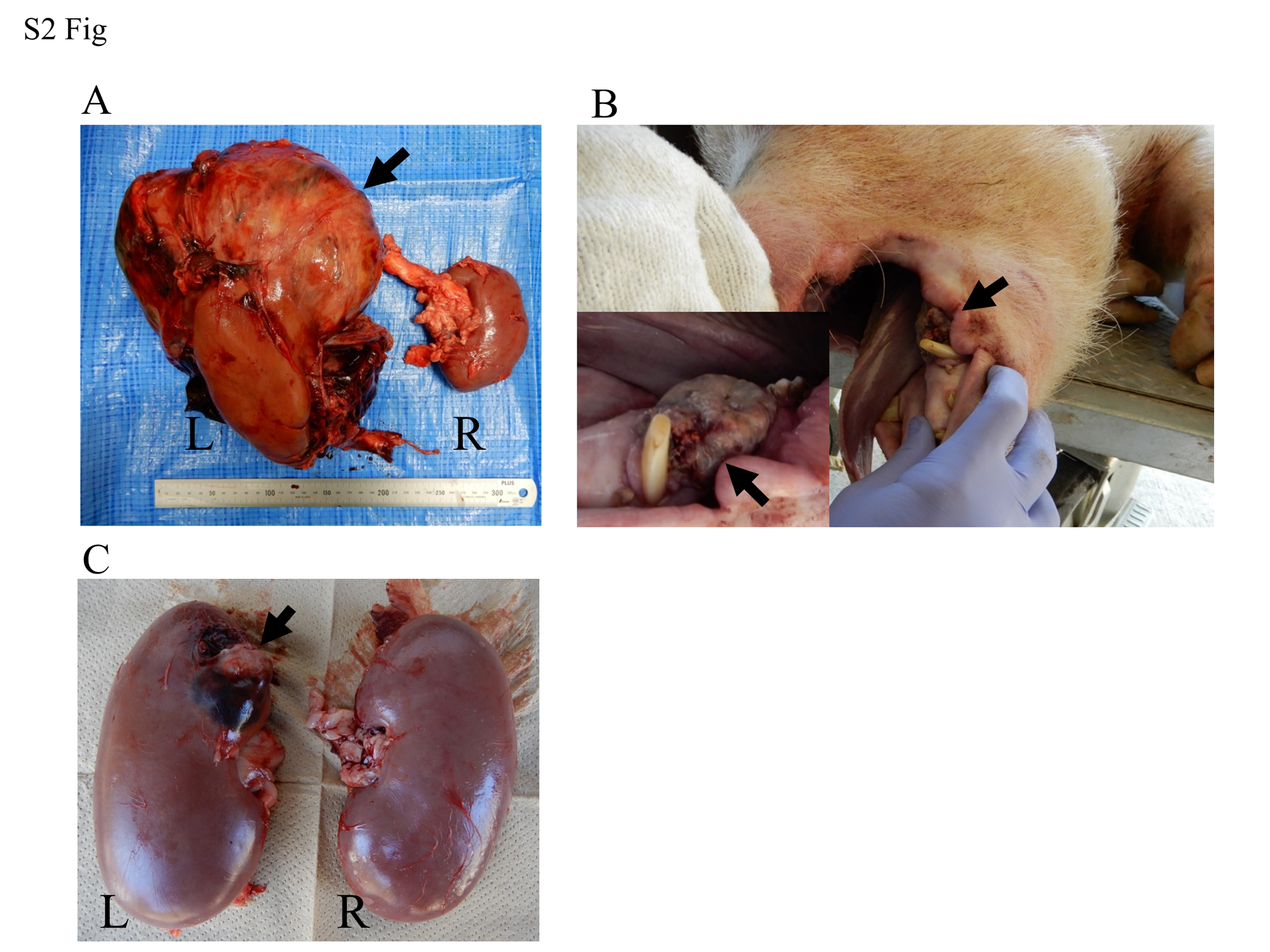

Supplement: S2 Fig — A: Large tumor mass of a nephroblastoma in the left kidney of pig #6 (arrow). L: Left kidney. R: Right kidney. B: Mandibular osteosarcoma (arrow) of pig #1. C: Nephroblastoma in the left kidney (arrow) of pig #1. L: Left kidney. R: Right kidney. (TIF) [file pone.0206360.s002.tif]
