# Supplementary material for: Generation of a TP53-modified porcine cancer model by CRISPR/Cas9-mediated gene modification in porcine zygotes via electroporation
Source: PLoS One. 2018 Oct 23;13(10):e0206360. doi: 10.1371/journal.pone.0206360 (PMC6198999; doi:10.1371/journal.pone.0206360)
Supplement: S1 Table — (DOCX) [file pone.0206360.s003.docx]

**S1 Table. Tumor marker serum levels in *TP53*-mutant pigs.**

| Piglet | Mutation | Indels | Frequency | Tumor marker* | Level | Normal levels in humans |
| --- | --- | --- | --- | --- | --- | --- |
| #1 | Bi-allelic | −7 bp | 14/14 | CEA (ng/ml) | <1.0 | <5.0 |
|  |  |  |  | TPA (U/l) | <3 | <75 |
|  |  |  |  | BFP (ng/ml) | 23 | <75 |
| #2 | Mosaic mutant | none | 1/11 | CEA | 1.0 | <5.0 |
|  |  | −7 bp | 9/11 | TPA | <3 | <75 |
|  |  | −13 bp | 1/11 | BFP | 60 | <75 |
| #4 | WT |  |  | CEA | <1.0 | <5.0 |
|  |  |  |  | TPA | <3 | <75 |
|  |  |  |  | BFP | 33 | <75 |
| #5 | Mosaic mutant | None | 3/13 | CEA | 1.0 | <5.0 |
|  |  | −142 bp | 6/13 | TPA | <3 | <75 |
|  |  | −151 bp | 4/13 | BFP | 740 | <75 |
| #6 | Bi-allelic | −4 bp | 2/15 | CEA | <1.0 | <5.0 |
|  |  | −7 bp | 4/15 | TPA | <3 | <75 |
|  |  | −4 bp/+5 bp, −15 bp | 9/15 | BFP | 27 | <75 |
| #9 | Mosaic mutant | none | 8/15 | CEA | 1.2 | <5.0 |
|  |  | +1 bp | 7/15 | TPA | <3 | <75 |
|  |  |  |  | BFP | 47 | <75 |

*CEA, TPA, and BFP are serum markers for general tumors. CEA, carcinoembryonic antigen; TPA, tissue polypeptide antigen; BFP, basic fetoprotein; WT, wild-type.
