# Supplementary material for: Generation of a TP53-modified porcine cancer model by CRISPR/Cas9-mediated gene modification in porcine zygotes via electroporation
Source: PLoS One. 2018 Oct 23;13(10):e0206360. doi: 10.1371/journal.pone.0206360 (PMC6198999; doi:10.1371/journal.pone.0206360)
Supplement: S2 Table — (DOCX) [file pone.0206360.s004.docx]

**S2 Table. Oligonucleotide sequences used to generate sgRNAs.**

|  | Forward oligos |  | Reverse oligos |
| --- | --- | --- | --- |
| sgRNA1 | TAGGTCTTCTGAGAAGGGACAA |  | AAACTTGTCCCTTCTCAGAAGA |
| sgRNA2 | TAGGTAAACAGAAAAACCCGGG |  | AAACCCCGGGTTTTTCTGTTTA |
